# Critical considerations for the practical utility of health equity tools: a concept mapping study

**DOI:** 10.1186/s12939-018-0764-6

**Published:** 2018-04-23

**Authors:** Bernadette Pauly, Wanda Martin, Kathleen Perkin, Thea van Roode, Albert Kwan, Tobie Patterson, Samantha Tong, Cheryl Prescott, Bruce Wallace, Trevor Hancock, Marjorie MacDonald

**Affiliations:** 1 Canadian Institute for Substance Use Research (CISUR), University of Victoria, PO Box 1700 STN CSC, Victoria, BC V8W 2Y2 Canada; 2 College of Nursing, University of Saskatchewan, 104 Clinic Place, Saskatoon, SK S7N 5E5 Canada; 3 British Columbia Ministry of Health, PO Box 9646 STN PROV GOVT, Victoria, BC V8W 9P1 Canada; 4 Centre for Chronic Disease Prevention and Health Equity, Public Health Agency of Canada, 1505 Barrington Street, Suite 1525, Halifax, NS B3J 3Y6 Canada; 5 Fraser Health Authority, Suite 400, Central City Tower, 13450 – 102nd Avenue, Surrey, BC V3T 0H1 Canada; 6 Clinical Prevention Services Education, BC Centre for Disease Control, 655 West 12th Avenue, Vancouver, BC V5Z 4R4 Canada; 7 School of Public Health and Social Policy, University of Victoria, PO Box 1700 STN CSC, Victoria, BC Canada

**Keywords:** Health equity, Tools, Concept mapping, Public health systems, Assessment criteria

## Abstract

**Background:**

Promoting health equity within health systems is a priority and challenge worldwide. Health equity tools have been identified as one strategy for integrating health equity considerations into health systems. Although there has been a proliferation of health equity tools, there has been limited attention to evaluating these tools for their practicality and thus their likelihood for uptake.

**Methods:**

Within the context of a large program of research, the Equity Lens in Public Health (ELPH), we conducted a concept mapping study to identify key elements and themes related to public health leaders and practitioners’ views about what makes a health equity tool practical and useful. Concept mapping is a participatory mixed-method approach to generating ideas and concepts to address a common concern. Participants brainstormed responses to the prompt “To be useful, a health equity tool should…” After participants sorted responses into groups based on similarity and rated them for importance and feasibility, the statements were analyzed using multidimensional scaling, then grouped using cluster analysis. Pattern matching graphs were constructed to illustrate the relationship between the importance and feasibility of statements, and go-zone maps were created to guide subsequent action.

**Results:**

The process resulted in 67 unique statements that were grouped into six clusters: 1) Evaluation for Improvement; 2) User Friendliness; 3) Explicit Theoretical Background; 4) Templates and Tools 5) Equity Competencies; and 6) Nothing about Me without Me- Client Engaged. The result was a set of concepts and themes describing participants’ views of the practicality and usefulness of health equity tools.

**Conclusions:**

These thematic clusters highlight the importance of user friendliness and having user guides, templates and resources to enhance use of equity tools. Furthermore, participants’ indicated that practicality was not enough for a tool to be useful. In addition to practical characteristics of the tool, a useful tool is one that encourages and supports the development of practitioner competencies to engage in equity work including critical reflections on power and institutional culture as well as strategies for the involvement of community members impacted by health inequities in program planning and delivery. The results of this study will be used to inform the development of practical criteria to assess health equity tools for application in public health.

## Background

The Ottawa Charter for Health Promotion explicitly identified equity and social justice as essential prerequisites for health, and equity in health as an important outcome of health promotion [1]. Since the Ottawa Charter, promoting health equity has been a priority for public health systems worldwide [2–4]. Starfield [5] described inequities in health as “systematic differences in one or more aspects of health status across socially, demographically, or geographically defined populations or subgroups” (p.546). Health inequities are unfair, avoidable, and potentially remediable; removing barriers to resources for health is essential for achieving health potential [6]. Health inequities are understood to be a consequence of unjust social structures that disadvantage some groups more than others [7].

There is support for, and agreement on, the need to improve health equity and strengthen the implementation and integration of health equity considerations in health care systems internationally [8–10]. Public health leaders have identified the use of health equity tools and resources as one promising strategy for doing this [11, 12]. Health equity tools have a range of purposes; they can help public health professionals assess the degree to which health equity considerations are included in policies or programs, or to measure health equity. Tools can also aid in choosing actions to address specific health inequities [13–15]. They can be used to: a) guide the conduct and interpretation of systematic reviews [16–18], b) incorporate cultural and gender considerations [19, 20], and c) conduct equity audits [21] or equity focused health impact assessments [22–26].

While health equity tools are meant to strengthen and integrate health equity considerations into public health policies, programs, and practices, there are challenges related to identifying, selecting, appraising and applying health equity tools [27]. Public health practitioners and decision makers struggle to apply a health equity lens in their work [28]. They identify that a lack of shared understanding of health equity and lack of guidance for applying health equity tools makes the use of tools challenging [29, 30]. Tyler et al. [28] found that when implementing an equity focused health impact assessment tool, factors at the system, organizational and operational level made using the tool a challenge. The main barriers to use of this tool include competing priorities; lack of organizational commitment, readiness and resources; and lack of shared understanding about health equity. In Canada, the National Collaborating Centre on Determinants of Health identified that the two most common challenges to health equity action were “getting support to take action on social justice issues in a health system based on biomedical principles” and “not being sure of what to do or what tools are available to help address health equity” [31].

So, despite the growing number of equity-focused resources, lenses, gauges, frameworks, and tools that span a range of purposes [27], there is little guidance in the literature to help practitioners and decision makers choose the appropriate tools, and we know little about the resources and supports that are needed to apply and use these tools. For example, conducting an equity-focused health impact assessment requires significant time and resources compared to using other tools that consist of brief checklists. To our knowledge no specific criteria have been developed for assessing health equity tools in relation to their practical utility. We sought to address this knowledge gap in this study.

As part of the ELPH program of research, we sought to identify available health equity tools and to develop theoretical and practical criteria for assessing them [32]. To date, we have developed an inventory of the health equity tools (see http://www.uvic.ca/elph), and identified a range of equity theories underlying approaches to health equity action through a review of the literature [33]. The identification of equity theories will guide us in developing the theoretical criteria which will be the focus of a subsequent paper.

We define a health equity tool as a document or resource that clearly identifies improving health equity as a goal and provides a set of steps, questions, or a framework that people can follow to achieve this goal [27]. In this paper, we identify key elements and themes that public health practitioners and leaders deem important in making health equity tools both practical and useful. These findings will inform the development of a set of criteria for judging practical utility of health equity tools. Developing both the practical and theoretical criteria, constitute the next stage of our project. In the study reported in this paper, we used concept mapping [34] to explore the ideas of public health practitioners about what makes a health equity tool both practical and useful, and to determine the feasibility and importance of the ideas generated.

## Methods

### Concept mapping

Concept mapping is a participatory mixed method approach to research [23, 24] that enables a group to describe ideas in response to a focused question, sort the ideas, rate them on specific criteria, and subject the data to multivariate analysis. The results are transformed into maps for visual representation and interpretation [35] which can then be used to guide planning, implementation and evaluation of action. Overall, there are six phases to the method: (1) preparing (2) generating ideas through brainstorming, (3) sorting and rating the ideas, (4) processing the data to produce maps, (5) interpreting the data and maps, and (6) using the maps. We obtained ethical approval for this study from the University of Victoria, the University of British Columbia, and from each of the five health authorities in British Columbia that were partners in the ELPH project and participated in the concept mapping study.

#### Phase 1: Preparing

Phase 1 consists of establishing a ‘focus prompt’ and choosing participants [36]. The focus prompt is a statement or question that participants respond to in Phase 2, and these responses become the concepts for mapping. For this preparatory phase, we convened a small working group of public health practitioners and decision-makers to generate some initial ideas. We then discussed the focus prompt with the entire team at an ELPH whole team meeting. The team comprises researchers, project staff, public health decision makers and practitioners. After much deliberation with team members, to generate concepts we chose the prompt: “*To be useful, a health equity tool should*...”

We invited ELPH team members to participate in the concept mapping exercise and posted invitations on the websites of organizations affiliated with team members. Because concept mapping is a mixed method approach, drawing initially on qualitative data through brainstorming and sorting, the logic of qualitative sampling guided the selection of a purposive sample. The main criterion in a purposive sample is that the participant has experience with the phenomenon under study and will be a good informant [37]. Participants included public health practitioners and decision makers from the BC Ministry of Health, and five of the six health authorities: Fraser Health, Interior Health, Island Health, Provincial Health Services, and Vancouver Coastal Health, as well as other associated organizations. Within their organizations, participants were responsible for implementing an equity lens in the public health system, specifically in relation to two core public health programs: mental health promotion and preventing substance use harm. Since the aim in qualitative research is not statistical generalizability, sampling is driven by a concern with the conceptual requirements of the study.

We asked e-mail recipients to forward the invitation to anyone they thought might be interested in participating. Although it was not possible to track the number of people that received the invitation, there was no need to do so because concept mapping does not limit the number of participants. Concept mapping was initially developed for 40 people or fewer when conducted face-to-face [34] and although a larger sample size potentially adds greater clarity to the results, there is a diminishing return as the number grows [38]. Therefore, response rate is not of great concern and not all participants need to be involved in every phase of the process. We used online Concept Systems software thereby allowing greater reach in terms of participation [39] because participants are spread across the vast geographic area of British Columbia. We incorporated the consent form into the online program with participants completing the consent before responding to questions.

#### Phase 2: Generating ideas through brainstorming

In Phase 2, we asked participants to generate as many ideas as possible to answer the focus prompt [36]. Forty-eight anonymous respondents contributed to this brainstorming phase, which occurred online using a secure site. Throughout the phase, all ideas generated were visible to all participants to allow one person’s ideas to spark another’s. A benefit of anonymous online participation is that everyone is free to make their statement without fear of criticism or controversy [36]. Participants generated 76 statements. The fact that all ideas were visible to everyone may have contributed to participants perceiving that their ideas had already been identified by others and so limited the number of statements each person contributed.

#### Phase 3: Sorting and rating ideas

In Phase 3, two team members (WM & KP) clarified the statements (e.g., correcting grammar, synthesizing ideas, deleting duplicates while staying as close as possible to the participant’s original wording) resulting in a final set of 67 unique statements. Additional team members then reviewed the statements and confirmed them. Using the online concept mapping software, participants were then asked to sort the 67 statements into themes or categories based on the similarity of ideas [36]. They also rated the statements from 1 to 5 on the dimensions of importance and feasibility, with 1 representing lower importance or feasibility and 5 representing greater importance or feasibility. In total, 26 people participated in the sorting process (19 completed) and 37 participated in the rating of importance and feasibility (30 and 27 respectively completed). Participant demographics are described in Table 1 for the 30 participants who completed either the sorting or rating process.

**Table 1:** Demographic characteristics of participants involved in sorting and rating

	Frequency (%) (*N* = 30)
Education	
Diploma	1 (3.3)
Baccalaureate	5 (16.7)
Masters	14 (46.7)
Doctoral	10 (33.3)
Position	
Full-time	24 (80.0)
Part-time	6 (20.0)
Setting	
Public Health	18 (60.0)
Other	12 (40.0)
Work Type	
Advocacy	1 (3.3)
Policy – front line	1 (3.3)
Policy – management	6 (20.0)
Practice – front line	3 (10.0)
Practice – management	9 (30.0)
Other	10 (33.4)
Work Area	
Business	1 (3.3)
Community development	1 (3.3)
Epidemiology	2 (6.7)
Health care administration	1 (3.3)
Health Promotion	2 (6.7)
Medicine	1 (3.3)
Nursing	10 (33.4)
Nutrition	1 (3.3)
Psychology	1 (3.3)
Public Health	4 (13.3)
Public Policy	2 (6.7)
Social Work	2 (6.7)
Other	2 (6.7)
Work involves substance use	
Yes	19 (63.3)
No	11 (36.7)
Work involves mental health	
Yes	20 (66.7)
No	10 (33.3)

#### Phase 4: Processing the data to produce maps

In Phase 4, we analyzed the data and produced the concept map, point-rating map, ladder graph, and Go-Zone maps. We used Concept Systems™ software [39] to generate all statistics and maps. Starting with information from the statement sorting, the program identifies two statements clustered together in the same category. On a matrix of N x N statements, the program assigns a 1 if the statements are placed together and 0 if not placed together, allowing for the total across all participants for each statement pair to have a number between one and the total number of participants [36]. The total similarity matrix is analyzed using non-metric multidimensional scaling (MDS) on two dimensions, allowing for representation on an XY axis called a point map [36]. Statements grouped together most often are closer together on the map. The program then uses the point map output in hierarchical cluster analysis that partitions the configuration into non-overlapping clusters in two-dimensional space (called a cluster map) [36]. The researchers decide on the number of initial clusters. At this stage in the analysis, the researchers must carefully consider the statements in each cluster to decide whether they are better grouped together or divided.

Starting with the 12 clusters identified in the Concept Systems output, we examined each group of statements to make sense of the grouping mindful of our participants’ original decisions to sort particular statements together. We merged clusters that contained similar sets of statements until we arrived at a final set of six clusters, and additional merging no longer made conceptual sense. For example, to reduce the clusters further from our current 6 to 5, cluster 6 statements that we have called ‘Nothing about Me without Me – Client Engaged’ would have been mixed with cluster 5 statements, called ‘Equity Competencies’. These sets of statements are very different conceptually and thus are best kept as two separate clusters. Discussions with participants in the whole team meeting confirmed their agreement that the clusters were distinct and the statements within them fit well with the overall meaning of the cluster as a whole, and thus made conceptual sense.

The software program showed the top ten cluster names assigned by the participants. In this same meeting, researchers and participants then reviewed the statements in each cluster, and selected the name for each that most closely described our shared understanding of why participants placed the responses together.

Additionally, the importance and feasibility ratings are averaged across participants for each item and for each cluster. This produces a point-rating map, which we generated for the entire set of statements and for each cluster. A ladder graph is used to compare the ratings for the importance of statements in a cluster to the ratings for the feasibility of statements in that cluster, giving a Pearson’s correlation coefficient (r) to describe the extent of the correlation [36]. A Go-Zone map uses the importance and feasibility ratings to produce a 2 × 2 table with the most important and most feasible statements or clusters in the top right hand quadrant.

#### Phase 5: Interpreting the data and maps

In Phase 5, researchers and participants interpret the maps. The closer that statements are located on the map, the closer they are conceptually. In other words, clusters of statements are used to structure ideas, producing what could be called a conceptual framework for the issue or problem. The Go-Zone map and ladder graph allow the relative importance and feasibility of statements and clusters to be assessed to indicate the most actionable areas on which to focus, and the areas of importance that may be more difficult to implement or of less importance.

#### Phase 6: Using the maps

Phase 6 is the final phase in which the maps are used for evaluation and planning; they are the result of collective thinking on a specific question. This paper represents a Phase 6 strategy for evaluation and planning.

## Results

### Cluster map

**Fig. 1:**
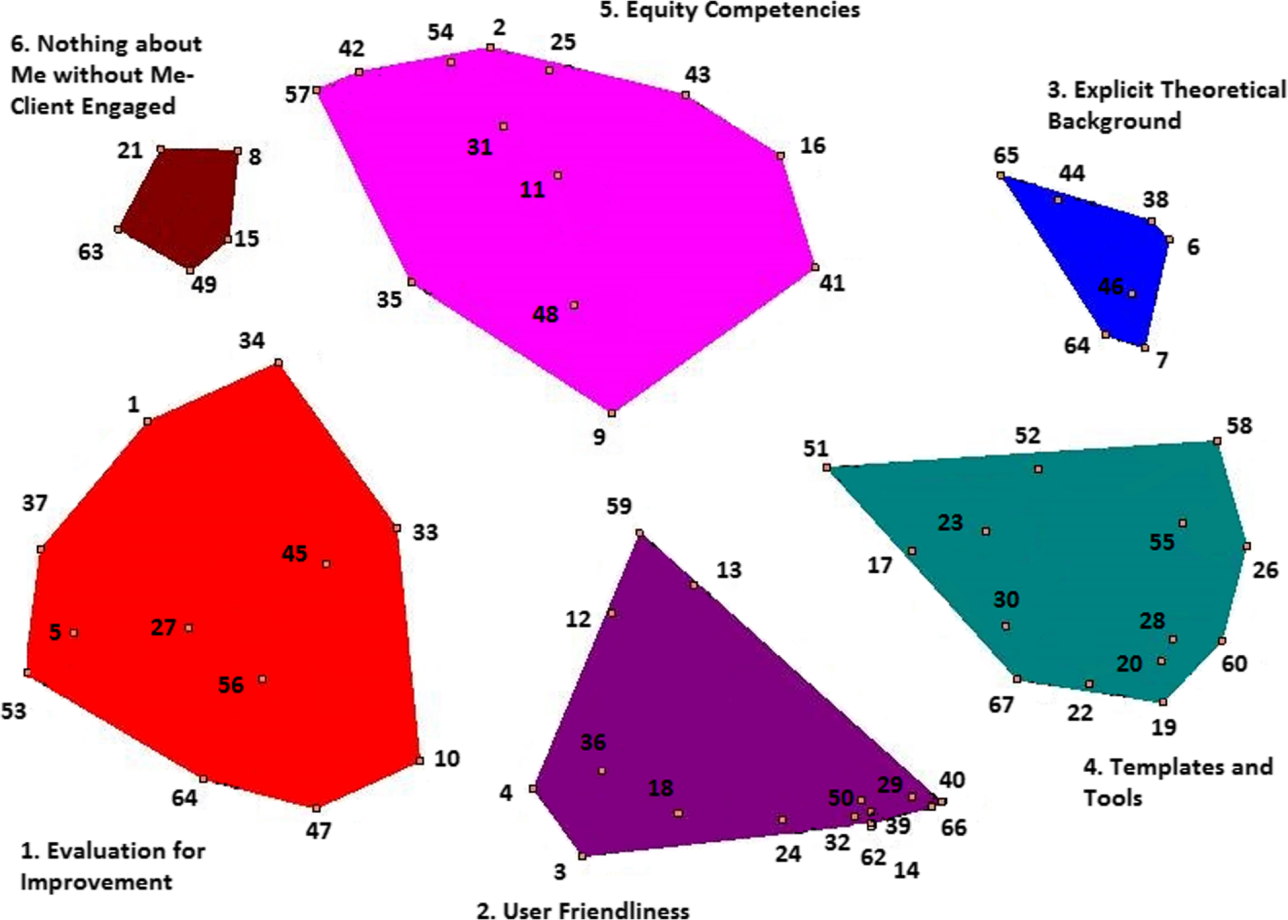
Cluster Map. Map displays which statements (by number) are contained in each cluster. Statement numbers correspond to statements given in Table 2

Participants generated 67 unique statements in response to the focus prompt “*To be useful, a health equity tool should*...”, and sorted them into clusters. After the MDS and cluster analysis, we identified a final set of six clusters. The specific statements in each cluster are listed in Table 2 and the final six clusters are presented in Fig. 1. The statement numbers in Fig. 1 correspond to the statement numbers in Table 2.

**Table 2:** Statements by Cluster to the focus prompt “To be useful, a health equity tool should…”

Cluster 1: Evaluation for Improvement	
1.	Be linked to Action Research
5.	Have a clear feedback loop to improve practice
10.	Have a clear intended outcome (e.g., is the tool intended to help you assess if there’s an inequity? To improve equity of an existing program? etc.)
27.	Assist program planners to improve the equity of their programs
33.	Provide further information or support after completing the tool, such as future steps and strategies to apply after identifying gaps or areas requiring attention
34.	Engage the client or patient in thinking beyond the individual to the social factors impacting health: consciousness raising or thought-inspiring.
37.	Lead to the identification of areas for improvement in policy/program
45.	Help define priorities
47.	Be useful for program evaluation
53.	Include a plan to evaluate after use
56.	Make sense to public health clients
64.	Be evaluated
Cluster 2: User Friendliness	
3.	Be applicable to a diverse range of situations and program areas
4.	Be a living document that can be updated following evaluations as to how the organization/workplace is doing to live up to a policy
12.	Guide your thought process
13.	Help the user to determine relevant strategies to address the inequity
14.	Be concise
18.	Have obvious relevance
24.	Have a clear purpose and objectives
29.	Be easy to understand
32.	Be short
36.	Be useful at various levels of the organization, front line work and policy making
39.	Be easy to use
40.	Be simple
50.	Be quick for a public health practitioner to use
59.	Give practical ways for the health care sector/providers to engage patient as full partner
62.	Be clear
66.	Use plain language
Cluster 3: Explicit Theoretical Background	
6.	Provide references for the theoretical foundations of the tool
7.	Have some context (e.g. background information)
38.	Provide an explanation of the theoretical foundations of the tool
44.	Be grounded in theories of health equity that illustrate how health inequities can be reduced
46.	Provide a clear definition of the fundamental principles of health equity (what is means, why it is important, practicalities, costs and limitations)
61.	Define equity
65.	Be grounded in theories of health equity that illustrate how health inequities occur
Cluster 4: Templates and Tools	
17.	Provide a way of synthesizing across the steps of the process to lead to a conclusion about what needs to be done
19.	Provide examples of how the tool can be used
20.	Describe appropriate applications of the tool
22.	Describe inappropriate applications for using the tool
23.	Provide links to tangible step-by-step strategies to act on any identified barriers to health equity. Ideally, this would be interactive with tailored suggestions based on assessment results
26.	Provide examples of how the tool has been used
28.	Provide a template or worksheets that can be completed by the user
30.	Provide a clear set of steps that help the user to determine whether a health inequity exists
51.	Provide guidance on determining strategies to address inequity
52.	Provide resources where the user can go from additional information or help
55.	Show how to work through the process of defining an inequity and determining strategies to address it
58.	Provide core sets of equity indicators
60.	Provide examples of how the tool could be used
67.	Clearly define appropriate context for use of the tool (organization-level policy assessment vs. front-line direct service program assessment)
Cluster 5: Equity Competencies	
2.	Extend the definition of culture to include how institutions may impact how people receive/experience care (i.e. religious upbringing, foster care, correctional institutions, street culture)
9.	Provide connections to a community of practice, or people to discuss health equity with
11.	Encourage the inclusion of harm reduction strategies to improve peoples’ health
16.	Be filtered through all the public health lenses
25.	Guide people through critical reflexivity exercise/mindfulness – how they show up to work, what things colour their lens of the world, how they may be a health care provider, but show up with their patient as a judge or minister
31.	Encourage compassion for both the health care worker and client
35.	Engage the provider in thinking beyond the individual to the social factors impacting health: consciousness raising or thought-inspiring
41.	Be grounded in quality improvement
42.	Explore how stigma from health care sector plays out in the services and supports we provide
43.	Take health literacy into account
48.	Be inclusive of users of programs
54.	Operate from a spirit of curiosity
57.	Take into account people’s trauma histories
Cluster 6: Nothing about Me without Me – Client Engaged	
8.	Include the participation of those affected by health inequities
15.	Point out ways that health care may be neglecting particular populations for more “favourable” populations
21.	Be inclusive of the health needs of people who use substances
49.	Feed courage to health care providers to be able to provide some level of care to anyone who walks through the door as being in the right place
63.	Encourage health care providers to examine how they can provide more culturally competent, trauma-informed, care

The stress index is the statistic that is reported in MDS analysis to indicate goodness-of-fit of the two-dimensional configuration to the original similarity matrix [34]. A low stress value suggests a better fit. Trochim [35] reported an average stress value of 0.285 across 33 studies, and approximately 95% of concept mapping projects have a stress value between 0.205 and 0.365. The stress value for this study, 0.294, is well within the expected range.

#### Cluster 1: Evaluation for improvement

Cluster 1 consists of 12 statements with a focus on evaluation and quality improvement to ensure that the use of a tool results in action and improvement in policies and programs. There should be clear benefits of applying a health equity tool such as identifying program improvements, health equity priorities, or engaging clients in thinking about social factors that may impact health. Use of the tool should improve implementation of health equity action through clear intended outcomes such as identifying inequities or improving equity in an existing program, identification of next steps or strategies linking to action research, or providing a feedback loop to allow refinement and ongoing improvements. Health equity tools should contribute to program evaluation and evaluation should be integral to the use of the tools to facilitate improvements in implementation.

#### Cluster 2: User friendliness

This 16-statement cluster reflects a very practical set of responses that describe how important ease of use is for practitioners. Practitioners stressed the need for simplicity: a tool should be concise, use plain language, be easy to understand, and be quick for practitioners to use. Furthermore, a tool should be applicable to a diverse range of situations. It should apply to various levels of the organization while having a clear purpose and objective. A useful tool needs to guide the thinking process, help the user determine relevant strategies, and offer practical ways for providers to engage with people. These findings suggest that practitioners want a tool that is not only easy to understand, but also one that is applicable throughout an organization in order for it to be practical. Obviously, using one tool versus several is simpler yet it is important to remember that tools have a range of applications and purposes so practicality may have to be weighed in relation to fit and purpose.

#### Cluster 3: Explicit theoretical background

This seven statement cluster is smaller, reflecting a distinct view that, to be practical, a tool should make explicit fundamental principles and theoretical foundations, including definitions and explanations of health equity. Overall, the statements in this cluster reflect the importance of clear and explicit explanations of the meaning, definition, and theoretical constructs that underpin the tool.

#### Cluster 4: Templates and tools

The Templates and Tools cluster has 14 statements describing practical resources to help a practitioner use the tool. Participants highlighted the importance of having access to descriptions of appropriate and inappropriate applications of the tool, examples of how to use it, and resources or links for additional information and/or implementation strategies. Participants emphasized the importance of having the necessary templates and guidance documents that outline, in a stepwise fashion, how to use the tool. This could be in the form of manuals but, more importantly, participants are looking for examples and illustrations of how the tool should be or has been used. Of note, participants explicitly highlighted the importance of directions for synthesizing across the steps in a tool, outlining processes that lead to conclusions about actions to take. These cluster statements reflect that, to be useful, a tool needs to include practical “how to” information about selection and use.

#### Cluster 5: Equity competencies

Thirteen statements in the Equity Competencies cluster reflect the view of participants that health equity tools, to be useful, require a practitioner to have health equity competencies and that these competencies should be encouraged by and through the use of a health equity tool. These competencies include the need for practitioners to be aware of and reflexive in their practice as to: a) broader structural conditions that produce inequities and move beyond thinking from individual to social factors that impact health; b) institutional culture and take account of histories of trauma; c) the persistent stigma that exists within health systems that can impact the delivery of health care; and d) potential health literacy issues for some clients. Other important competencies include having compassion, and using harm reduction strategies to improve health. A health equity tool needs to support the development of competencies in applying a health equity lens to all public health programs, and facilitate connection to a community of practice. Overall, for health equity tools to be useful, they should incorporate and encourage the enactment of equity competencies.

#### Cluster 6: Nothing about me without me – Client engaged

As the smallest cluster with only five statements, this set of statements focuses on the need for health equity tools to promote inclusion of those populations impacted by health inequities such as people who use substances or those who are neglected relative to others. Tools should encourage practitioners to engage all such groups in a meaningful way so that the care delivered will be culturally appropriate and trauma-informed, as necessary.

##### Statements ratings and go zone map

**Fig. 2:**
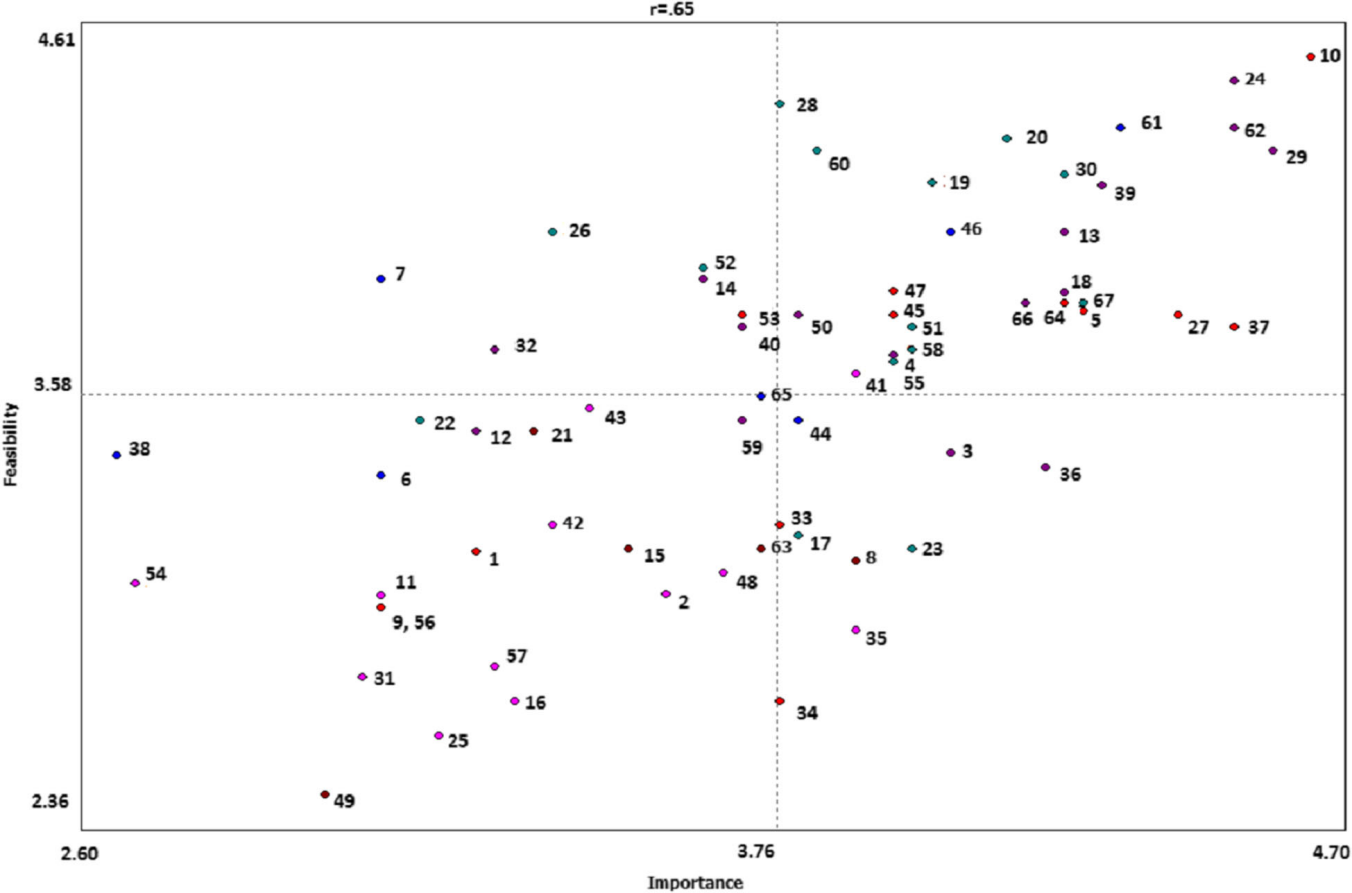
Go Zone Map of Mean Importance and Feasibility Ratings for All Statements. Ratings range from 1 to 5, with 1 indicating a low importance or feasibility, and 5 indicating high importance or feasibility. Statement numbers correspond to statements in Table 2

Subsequent to generating and clustering statements, participants rated each statement on importance and feasibility. We then averaged the ratings across participants for each statement. The Go Zone map in Fig. 2 compares the mean importance and feasibility for each statement, with the numbers on the map corresponding to the statement numbers in Table 2. Statements rated high in both importance and feasibility would represent potential priority areas of action. The top four statements that are both highly important and most feasible illustrate the need for a tool to be clear, easy to understand, and well defined, with a clear intended outcome, purpose and objectives. Statements that were rated highly important but less feasible include engaging both the provider and client to think beyond the individual to the social factors that impact health, and to include the participation of those affected by health inequities.

##### Cluster ratings and ladder graph

**Fig. 3:**
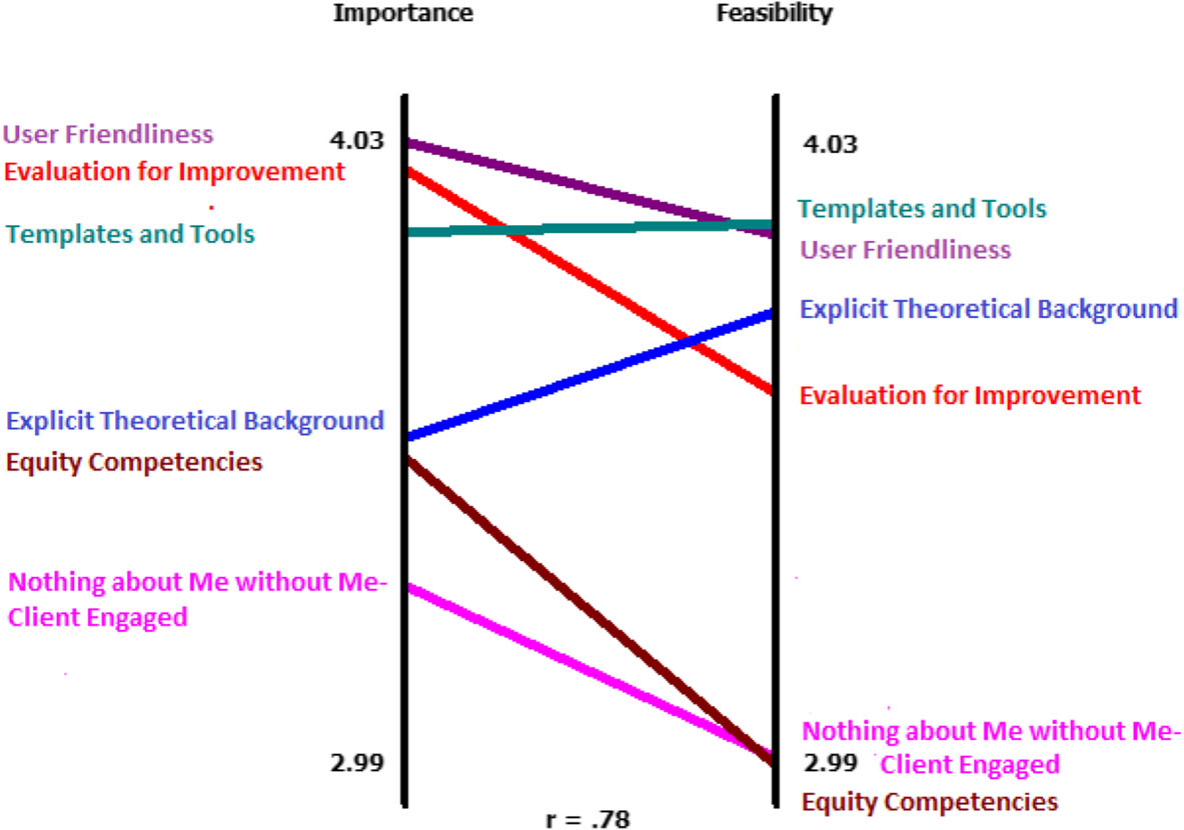
Ladder Graph of Mean Importance and Feasibility by Cluster. Ratings range from 1 to 5 with 1 indicating low importance or feasibility and 5 indicating high importance or feasibility. Placement of cluster name on each axis indicates order of importance and feasibility. Colour of cluster name corresponds to colour of the line on graph which gives the value for importance and feasibility. Pearson’s r reports the overall correlation between importance and feasibility ratings

In Table 3, we present the mean importance and feasibility for the statements in each cluster, and compare these in the ladder graph in Fig. 3. The greater the angle between the two clusters, the greater the difference on the scale. The most important and feasible clusters were User Friendliness and Templates and Tools. The strong correlation between importance and feasibility ratings (*r* = 0.78) indicates that there would be a good benefit in targeting the most feasible clusters as they were also rated highly important. Notably, all of the clusters were rated above 3 in both importance and feasibility indicating that there were no clusters that participants considered to be of low importance, or too difficult to address.

**Table 3:** Cluster mean ratings for importance and feasibility

Cluster Name	Importance	Feasibility
Evaluation for improvement	3.98	3.61
User friendliness	4.03	3.87
Explicit theoretical background	3.53	3.74
Templates and tools	3.88	3.89
Equity competencies	3.34	2.98
Nothing about Me without Me- Client engaged	3.49	3.02

## Discussion

Health equity tools have been identified as one strategy to increase health equity considerations in public health programs and the health sector more broadly. Despite a proliferation of health equity tools, there has been limited critical analysis of these tools, or assessment of their practical utility. Here we used concept mapping to understand what public health practitioners see as important characteristics of a useful health equity tool. This resulted in the construction of six clusters for consideration when developing, selecting and using health equity tools. Ratings of the importance and feasibility of the characteristics in these clusters showed that participants considered all clusters to be important and comparatively feasible to implement, and that targeting the most feasible of the clusters would be strategic because they were also rated as highly important.

A key aspect of these findings is the importance of having tools that have clear and actionable objectives and cycles for evaluation, explicit theoretical foundations, are user friendly, and have supporting guidance documents available (Clusters 1–4). Although some of the statements in these clusters are specific to health equity, these higher level themes demonstrate understanding of what is needed for successful development and delivery of programs in general. Some of these findings are similar to those of Guichard, et al. [40], who used concept mapping to prioritize and identify important conditions for implementing a specific health equity tool (GAALISS tool) in public health practice. These conditions included specifying necessary characteristics of the tool being implemented such as: the tool should be short, have simple terminology, be accompanied by examples, and have a user guide. Their work, and that of Tyler et al. [28], highlighted the importance of leadership, organizational priorities and readiness, and intersectoral partnerships as important conditions for implementing both the GAALISS and Health Equity Impact Assessment (HEIA) health equity tools. That these conditions did not emerge in our work is likely a result of our focus prompt. Participants may have assumed that conditions were already in place, with our participants focusing on the process of implementing a tool as part of their practice and within their sphere of influence. As well, in our work, we identified the importance of having tools that have explicit and coherent theoretical foundations.

Our findings do speak to the value of community engagement and empowerment, as well as the importance of reflexivity and specific knowledge of the root determinants of health inequities in the process of implementing health equity tools (Cluster 5). Others, examining the use of knowledge to action frameworks as important strategies for improving action on health equity [41, 42], have highlighted similar findings. Masuda et al. [43] specifically stress the importance of critical inquiry and reflexive practice as part of the third wave of knowledge translation in terms of moving from ‘what we know’ to ‘how we know’, and recognizing the underlying power relations that shape knowledge in order to promote a more fair and just world within our spheres of influence. McCalman et al. [44] reviewed Indigenous health promotion tools, and these authors also emphasize the importance of cultural competency, community engagement and empowerment, and capacity development as well as partnerships, holism, best practice, and sustainability as key elements.

Our participants identified that to be useful equity tools should encourage the development and use of equity competencies as important to practicality. Some might argue that these elements go beyond the characteristics of the tool itself. It is important to remember, however, that our focus prompt did not ask specifically for characteristics of tools. Rather, it asked “To be useful, a health equity tool should……”. This prompt essentially asked participants about what a health equity tool should be or do in order to be useful. In the view of our participants, a useful tool is not just one that is easy to use, or has a clear purpose or objectives. It is one that encourages practitioners to think in a particular way, and to have a way of being that reflects the values and principles of health equity.

In relation to Cluster 5 (equity competencies), participants specifically highlighted the importance of being aware of stigma and the importance of harm reduction as key elements that make a health equity tool useful. This is not surprising given that 2/3 of participants reported working in public health programs related to mental health promotion and preventing the harms of substance use. Harm reduction is an evidence based non-judgmental approach that focuses on preventing harms of substance use while emphasizing respect, dignity, compassion and meaningful inclusion without expecting cessation of use [45]. Such an approach is clearly consistent with principles of health equity in relation to substance use programs [46].

Cluster 6, which focuses on client engagement in the use and application of health equity tools, highlights key social justice perspectives [47] that call for meaningful involvement and participation of those affected by health inequities in the application and use of health equity tools. Community participation is a central plank in health promotion [1]. In fact, the very naming of that cluster, Nothing about Me without Me- Client Engaged, reflects a popular and well known saying, both in Canada and elsewhere, that builds on original work in disability studies involving people with lived experience in issues that impact their lives [48].

As such, Clusters 5 and 6 specifically highlight public health commitments to social justice, and speak to the importance of developing critical consciousness in relation to structural and systemic injustices that have produced health inequities for those facing structural disadvantages due to gender, ethnicity, sexual orientation, class, ability and how they are positioned within current systems of care [49, 50]. It seems reasonable to us that our participants identified these aspects of public health and social justice as key elements in determining the usefulness and practicality of health equity tools. These are the very foundations of public health [51, 52]. If a tool does not support practitioners to work in practical and meaningful ways with people impacted by health inequities, then practitioners did not view it as useful to their practice. Although these clusters reflect a unique perspective of practitioners focused on health equity work in the area of mental health promotion and preventing harms of substance use, we would argue that these clusters may be applicable to judgements about the usefulness of health equity tools in other areas of public health practice. Specifically, we highlight important practitioner insights about the need for tools that encourage individual reflexivity on power and position and recognition of how stigma embedded in institutional cultures can obscure the structural and social conditions that impact health. We note that the overall sample contained participants in a wide range of roles from each health authority, all with varying levels of responsibility and education. Thus these viewpoints are from people with experience at different levels of authority and from program areas of the organization.

Strategic direction and action planning can occur with the use of Go-Zone maps and ladder graphs [34]. The clusters entitled ‘User Friendliness’ and ‘Templates and Tools’ indicate that having a clear and well defined tool is both highly important and feasible. For designers of tools, ensuring that a tool is clear and concise, with sufficient examples and information to aid in the selection and use of the tool are comparatively simple areas to target that are highly important in ensuring a tool’s utility. We would add that making a tool clear and concise does not necessarily mean that it reflects a sound theoretical understanding of health equity. Given previous research on the lack of shared understanding of health equity among public health leaders and practitioners, and the difficulties in applying a health equity lens to take action on promoting health equity [28–30], it seems insufficient and restrictive to focus only on the form of the tool, such as making it simple, concise and easy to use.

Statements and clusters ranked as important but less feasible offer areas for reflection and deliberation on ways that guidance in these clusters could be achieved. Participants noted that to be useful, application of health equity tools should be complemented by the development of practitioner competencies related to health equity. These competencies include taking a reflexive position and possessing a compassionate understanding of the broader structural injustices that produce health inequities, and being able to raise practitioner consciousness about the roots of health inequities. Participants also noted the importance of tools that promote inclusion and meaningful involvement of clients to address health inequities. Although these areas may be more difficult to address, these clusters draw attention to important elements of a health equity tool that need to be considered beyond the form of the tool itself in order to make it truly useful and avoid unintentionally perpetuating health inequities and/or harms. A strength of this study is that it directly involved public health leaders and practitioners in considering an issue that is relevant in their work. The fact that all of the clusters had comparatively high importance and feasibility indicates the careful consideration that went into the generation of all the responses as to what makes a useful health equity tool, and that all clusters need to be considered in the development of practical criteria.

## Conclusion

Our public health partners identified challenges in identifying and applying a health equity lens and tools, and were lacking guidance on how to work effectively toward reducing health equities. Thus participants were well situated to provide insight into what would make a useful health equity tool from their experience with searching for, selecting, and using such tools. Overall, few studies have addressed the theoretical or practical aspects of selecting and implementing health equity tools as an intervention to promote action on health equity. In this paper, we have identified and described clusters of thematic elements that can inform the development of practical criteria for constructing, selecting and using health equity tools. The findings of this study provide the basis for these yet-to-be developed criteria. In addition to the obvious characteristics of health equity tools that reflect practicality such as being useful in evaluating and informing improvements, being user friendly and including tools and templates to support use, our participants also identified unique aspects of usefulness. That is, to be useful, health equity tools need to have explicit theoretical foundations, promote health equity competencies that are rooted in critical conceptions of social justice and ensure that practitioners engage and participate directly with community members who are experiencing health inequities. These aspects of usefulness have important implications for public health systems in supporting health equity action in organization.

## Abbreviations

ELPH: Equity Lens in Public Health
HEAT: Health equity assessment tool
HEIA: Health Equity Impact Assessment
MDS: Multidimensional scaling
